# p75^NTR^ in Huntington's disease: beyond the basal ganglia

**DOI:** 10.18632/oncotarget.6646

**Published:** 2015-12-17

**Authors:** Verónica Brito, Silvia Ginés

**Affiliations:** Departament de Biologia Cellular, Immunologia i Neurociències, Facultat de Medicina, Universitat de Barcelona, Barcelona, Spain; Institut d'Investigacions Biomèdiques August Pi i Sunyer (IDIBAPS), Barcelona, Spain; CIBERNED, Spain

**Keywords:** Huntington's disease, cognitive deficits, hippo-campus, p75 receptor, RhoA

Huntington's disease (HD) is a fatal neurodegenerative disorder with a characteristic phenotype including chorea and dystonia, uncoordinated fine movements, cognitive decline and psychiatric disturbances. Even though the clinical diagnosis of HD relies on the manifestation of motor abnormalities, the associated memory impairments have been growing in prominence. Indeed, cognitive deficits are evident along all the disease process even in the prodrome before any motor diagnosis is given.

These clinical signs have been mainly attributed to corticostriatal dysfunction being deficits of neurotrophic support, caused by either reduced levels of brain-derived neurotrophic factor (BDNF) [1] or decreased TrkB and aberrant p75^NTR^ signalling [2, 3], one of the major pathogenic mechanism involved. However, in recent years has emerged the idea that cognitive decline in HD is likely a reflection of a widespread brain circuitry defect rather than a basal ganglia dysfunction *per se*. In this regard, in our recent studies we shed new light on the contribution of the hippocampal circuitry to synaptic and memory decline in HD. We demonstrated hippocampal dysfunction in a precise genetic HD mouse model that expresses endogenous levels of mutant huntingtin, Hdh^Q7/Q111^ mice, manifested as alterations in spatial, recognition and associative memories.

To get insight into the molecular mechanisms underlying such defects, we focused on p75^NTR^ since growing evidence indicate that p75^NTR^ plays an antagonistic role in synaptic plasticity. We demonstrated up-regulation of p75^NTR^ in the hippocampus of distinct HD mouse models and in human brain without evident changes in the cortex, which extends our previous data showing increased p75^NTR^ expression in the HD striatum [4]. In agreement with a critical role of aberrant p75^NTR^ expression in hippocampal dysfunction we found preserved spatial, recognition and associative memories in new double-mutant mice expressing mutant huntingtin but “*physiological”* levels of p75^NTR^ levels (*Hdh^Q7/Q111^:p75+/−*mice).

How aberrant p75^NTR^ levels may mediate synaptic and memory deficits in HD is an intriguing question. On one hand, our results indicated that p75^NTR^ directly or indirectly regulate the expression of different synaptic-related proteins previously implicated in HD synaptic and/or cognitive deficits, such as CBP, CamKII, GluA1 or BDNF since memory improvements in double mutant *Hdh^Q7/Q111^*:p75*^+/−^*mice correlated with a recovery of the expression and/or phosphorylation of these molecules. On the other, the loss of dendritic spines in CA1 pyramidal neurons exhibited by *Hdh^Q7/Q111^* mutant mice was also prevented by normalization of p75^NTR^ levels. Altogether, these data suggest that synaptic and memory deficits in HD could be related with a reduction in proteins involved in synaptic function and in the number and complexity of hippocampal dendritic spines in agreement with a role of p75^NTR^ as a negative regulator of dendritic spine dynamics and synaptic activity.

We further built on work showing that p75^NTR^contributes to synaptic dysfunction and memory decline in HD by deregulation of RhoA activity, a small GTPase with complex effects on spines and thereby in synaptic plasticity [5]. Interestingly, such increase was reversed in double mutant *Hdh^Q7/Q111^:p75^+/−^*mice suggesting a direct link between aberrant p75^NTR^ activity, dendritic spine loss and aberrant RhoA activity. In agreement with our findings Plotkin and colleagues [3] have demonstrated that plasticity in indirect pathway spiny projection neurons (iSPNs) from BACHD mutant mice can be rescued by inhibition of the p75^NTR^-RhoA signalling suggesting that early corticostriatal dysfunction in HD could also be attributable to a correctable defect in BDNF signalling. Altogether, this evidence strongly suggests that, in the early stages of the disease, p75^NTR^ antagonism should be considered an effective therapeutic strategy for restoring BDNF neuroprotective and synaptic functions. However, remains to be investigated whether therapeutic approaches targeting p75^NTR^ inhibition should also integrate TrkB activation to produce the most favourable benefits on motor and cognitive impairments.

Moreover, we have recently demonstrated that fingolimod, a compound used as an immunomodulator in Multiple Sclerosis patients, restores hippocampal synaptic plasticity and improves memory function in a mouse model of Huntington disease acting through down-regulation of TNFα and p75^NTR^ [6]. These last evidence open the question whether p75^NTR^ dysregulation is triggered by neuroinflammation at very early stages and whether modulation of the inflammatory response of microglia and astroglia in HD may contribute to antagonize the deleterious effects of aberrant p75^NTR^ induced RhoA activity in synaptic plasticity. So far, our studies strongly implicates p75^NTR^-RhoA hippocampal dysfunction on cognitive decline in HD stressing the need to explore therapeutic strategies that target the identified pathways, not only in the striatum but also in other brain areas besides the basal ganglia which has been underestimated and are strongly involved in HD cognitive pathology
